# Support and Emotional Well-Being of Asylum Seekers and Refugees in Spain

**DOI:** 10.3390/ijerph17228365

**Published:** 2020-11-12

**Authors:** Ruth León-Pinilla, Ana Soto-Rubio, Vicente Prado-Gascó

**Affiliations:** 1 Department of Translation and Intercultural Communication, Faculty of Social Sciences, European University of Valencia, 46021 Valencia, Spain; ruth.leon@universidadeuropea.es; 2 Department of Personality, Psychological Assessment and Treatments, Faculty of Psychology, University of Valencia, 46021 Valencia, Spain; ana.soto@uv.es; 3 Department of Social Psychology, Faculty of Psychology, University of Valencia, 46021 Valencia, Spain

**Keywords:** refugees, asylum-seekers, well-being, Spain, sociodemographic profile, linguistic knowledge

## Abstract

Although the world’s forcibly displaced population reached 79.5 million in 2019, their difficult situations and the issues they struggle with remain practically invisible in Spanish society. Therefore, it seems necessary to provide greater insight into an invisible reality to improve the refugees’ situation. The present cross-sectional study aims to draw a general profile of refugees’ and asylum seekers’ main characteristics in Spain and their well-being. A total of 186 refugees living in Spain participated. An ad-hoc questionnaire was administered to obtain data regarding sociodemographic profile, language skills, and social and institutional support. A standardized instrument, SPANE, was used to measure well-being. It can be seen that healthcare, followed by legal aid, are the easiest to access. On the other hand, finding a job, having money, and finding housing are the most difficult. In general, it seems possible to say that the refugees present more positive feelings than negative ones, which implies a positive emotional balance, although the average score obtained for emotional balance is quite far from the highest possible score. We consider this to be a pivotal first step which can provide useful information for the further design of aid strategies to improve this vulnerable group’s situation.

## 1. Introduction

The world’s forcibly displaced population grows every year worldwide [1]. Nevertheless, the difficulties faced by this group once they arrive in their host country remain practically invisible to general society (their lived reality and the issues they struggle with just to live are barely present in the media and political debates). This situation is so even though the group comprised of refugees is probably one of the most vulnerable among all migrant and minority groups [2], and that Article 14 of the Universal Declaration of Human Rights [3] sets forth that the right to seek asylum is an international human right.

In this context, it is necessary to gain greater insight into the current reality of refugees in different countries in order to enhance this group’s visibility and to raise awareness of the need to safeguard their human rights, favoring at the same time their successful integration into the new contexts that take them in [1].

### 1.1. Concepts and Definitions

There is a great deal of confusion among the media and society regarding the terms used to describe various profiles of forcibly displaced migrants [1,4,5,6]. In order to bring some clarity on this matter, definitions of the terms “refugee” and “asylum-seeker” as used in this paper are provided below:

The primary and universal definition of a refugee that applies to states is contained in Article 1(A) [2] of the 1951 United Nations Convention Relating to the Status Refugees, as amended by its 1967 Protocol [1], defining a refugee as someone who:

“owing to a well-founded fear of being persecuted for reasons of race, religion, nationality, membership of a particular social group, or political opinion, is outside the country of his nationality and is unable or, owing to such fear, is unwilling to avail himself of the protection of that country; or who, not having a nationality and being outside the country of his former habitual residence as a result of such events, is unable or, owing to such fear, is unwilling to return to it.”

This term is often confused with asylum-seeker, which refers to:

“a person who has left their country of origin and formally applied for asylum in another country but whose application has not yet been concluded” [7].

Any person susceptible of being deemed as a refugee and who finds themselves at some point of the process of being granted refugee status or international protection will be referred to as an asylum-seeker and a refugee (AS&R) in this paper. Therefore, asylum-seekers and refugees will be considered in conjunction in this study because, although these two categories have differences (whether a person is defined as a refugee or an asylum seeker determines the help, support, aid, and rights they have access to), they also have commonalities since they are forcibly displaced people: the challenges they have to overcome to get their basic needs covered, the culture and language-related difficulties, and the impact that this situation has on their well-being.

### 1.2. UNHCR and Other Entities that Work to Defend Refugees

In international terms, the most important organization that works with refugees is the United Nations High Commission for Refugees (UNHCR), which has the mandate to protect refugees and to find permanent solutions to the problems related to this issue around the world [1].

Specifically in Spain, the leading organization addressing the situation of refugees is the Spanish Commission to Help Refugees (CEAR), who states that their mission is to defend and promote Human Rights and the physical, emotional, and social health of refugees, stateless persons, and migrants who need international protection or are exposed to social exclusion [8]. In conjunction with the CEAR, other entities that address the needs of refugees are the Spanish Red Cross, four hosting centers for refugees supervised and financed by the Directorate General of the Integration of Immigrants, the Spanish Refugee and Asylum-seeker Defence Committee (COMRADE), Karibu (Friends of African People), International Rescue (Rescate Internacional) and the Spanish Catholic Association Commission for Migration (ACCEM).

The abovementioned social organizations play a pivotal role along the different stages of the asylum-seeking process, implementing a variety of programs and providing different kinds of aid, including legal and social assistance; medical and psychological care; support to families during the schooling process of minors; essential services; pastoral aid; and intercultural and interlinguistic mediation [9,10,11].

### 1.3. Refugees in Figures

By the end of 2019, UNHCR [1] had registered 79.5 million people who had been forcibly displaced throughout the world due to persecution, conflicts, generalized violence, or human rights violations. This cipher included 26 million refugees, of which 4.2 million were asylum-seekers, and 40% were under the age of 18.

Developing countries hosted 85 percent of the world’s refugees under UNHCR’s mandate, about 16.9 million people. The least developed countries provided asylum to a growing proportion, amounting to one-third of the global total (6.7 million refugees) [1]. More specifically, when focusing on the main recipient countries of the refugee population, it can be seen that Turkey hosted the largest number of refugees relative to its national population, hosting 3.6 million refugees. Colombia ranks second, with 1.8 million hosted refugees. Pakistan and Uganda hosted 1.4 million refugees each, and finally, Germany ranks fifth, with 1.1 million refugees.

Concerning the origin of the people seeking asylum, Syria remained the main country of origin of refugees at the end of 2019. More than 6.6 million people have been forced to flee the country, accounting for almost one-third of the world’s total refugee population [1]. The second-largest population of refugees in 2019 was from Venezuela (3.7 million people), followed by Afghanistan (2.7 million people), South Sudan (2.2 million people) and Myanmar (1.1 million people).

In 2019, Spain received more international protection applicants than any other year since the approval of the first Asylum Law in 1984, receiving a total of 118,264 applications [12]. Having granted refugee status to 5.2% of these applicants, Spain is far below the European Union average in this regard, which grants this status to 31% of the applications it receives [12].

### 1.4. Well-Being and Refugees

Refugees worldwide suffer high levels of distress [13]. Existing research [14,15,16,17,18,19,20] has shown that immigrants are a particularly vulnerable group to psychological disorders. Nevertheless, rates of help-seeking for mental health problems are low among refugee communities [21].

In this line, the most vulnerable group among immigrants is that of refugees, who, due to the circumstances surrounding the abandonment of the country of origin, are more vulnerable to mental health problems [22] and have to face many difficulties to integrate into the host country [2,23]. As described by Tribe [24], the circumstances in which they leave their countries are very different between the two groups:

“Refugees differ from immigrants in that the latter have usually made a positive choice to change their country of residence and have been able to plan the move practically, psychologically, and systematically over time. On the other hand, refugees usually have to flee for fear of their lives at short notice, often to unknown destinations. It is not a decision taken lightly, to risk losing everything your life has been built upon. Thus, to flee to a destination where an unknown future awaits can never be an easy decision. Nevertheless, refugees may also feel immense relief that they have reached a safe haven and know that their lives are no longer under threat” [24].

According to Tribe [24], refugees leave their place of origin due to compelling causes, such as “war, human rights abuses, persecution for political, religious, gender, or ethnic reasons. This flight leads to significant losses in the lives of these people, such as the loss of their country, culture, family, profession, language, friends, future plans, etc.” [24]

In addition to having to escape from their old life, the refugee has to face many problems related to basic needs, such as finding a safe place to live, water, food, communication and clothes [25]. They also have to face problems related to their social situation due to violent separation from their family, friends, environment and any feelings of loss and despair generated by that situation [26].

Refugees also have to face great difficulties related to their legal situation, dominated by a feeling of permanent uncertainty due to the very long time it takes to resolve the asylum request (minimum 6 months, up to 3 years, and even more). Furthermore, they have to face social inclusion problems due to difficulties in finding a job, the language barrier, economic difficulties, cultural difficulties and differences, social exclusion and housing needs [26].

Regarding the issues faced by refugees in the host country, Tribe [24] points out the situation of permanent change, psychological and practical adaptation, uncertainty about the future, traumatic events in their lives, extreme difficulties, racism, stereotypes projected from the host community and unknown cultural customs.

Likewise, several studies have been conducted in Spain suggesting that the lowest levels of life satisfaction and subjective well-being are among the immigrant population. The research positions itself differently in terms of the factors affecting this reality: demographic factors, such as country of origin, linguistic affinity, educational level, length of residence, work situation, etc. [27,28,29,30,31]; and social and contextual factors, such as social support, integration and the feeling of belonging to the community or social participation [31,32,33,34,35,36]. The general socio-economic context and characteristics of the asylum, the increase of rental housing and the requirements for the signing of a contract, the difficulties in accessing a job in dignified conditions, the uncertainty that accompanies the long and sinuous instruction of the application for international protection or the obstacles to accessing social assistance of regional scope define a very complex horizon for inclusion in Spain [12].

### 1.5. The Asylum Process in Spain

#### 1.5.1. The Status of Refugee

Spain’s Regulatory Act 12/2009 on the Right to Asylum and Subsidiary Protection in Spain [37] accords refugee status to anyone with a justified fear of persecution in their own country for reasons of race, religion, nationality, political views, or belonging to a specific social group, gender or sexual orientation. It also recognizes as stateless any person who has no nationality, is outside their country of habitual residence, and either does not wish to or is unable to return to that country for any of the above reasons.

The asylum process is an administrative procedure aimed at identifying people needing international protection from among claimants. It is based on the definition of ‘refugee’ as outlined in the Geneva Convention of 1951. According to the provisions set forth in Article 13 of the Spanish Constitution and international law following the Geneva Convention, the Spanish legislation establishes the conditions under which third-country nationals and stateless persons may obtain international protection in Spain [38]. International protection is granted by obtaining the status of refugee or through subsidiary protection, for which there is a single procedure: the claim for asylum.

The aforementioned claim is vital for the person who seeks it since a system of legal protection and welfare benefits is put into place if it is granted, thereby allowing that person to start a new life [39]. Article 6 (1) of Act 12/2009 [37] sets forth that, for a person to be granted the right to asylum, the actions on which the well-founded fear of being subject to persecution for reasons of race, religion, nationality, political opinion, membership of a particular social group, sex or sexual orientation “have to be sufficiently serious in their nature or reiteration” or “be a sufficiently serious accumulation of various measures, including human rights violations.”

As provided for by Article 10 of Act 12/2009 [37], when the right to subsidiary protection is granted, there are several circumstances which constitute serious damages, such as the death penalty, torture, and inhuman or degrading treatment in the country of origin, or serious threats against the life or integrity of civilians caused by indiscriminate violence in situations of international or internal conflict.

#### 1.5.2. The Granting of International Protection in Spain and Its Repercussions Regarding the Claimant’s Rights and Obligations

Spanish law sets forth the repercussions involved in the claim for asylum for whoever files such a claim. In particular, Article 19 of Act 12/2009 [37] establishes that the person who has claimed protection may not be subject to return, refoulement, or expulsion until his or her claim is resolved. He or she may not be extradited. He or she is entitled to be counseled by a lawyer and, should the claim’s processing exceed six months, the claimant has to be given notice of the reasons for the delay.

Likewise, Article 36 of Act 12/2009 [37] sets forth that the granting of the right of asylum or of subsidiary protection shall involve recognizing the rights established by the Geneva Convention Relating to the Status of Refugees, as well as in prevailing legislation on foreign nationals and immigration and in European Union legislation. These rights include: (a) protection from refoulement; (b) access to information in a comprehensible language on the rights and obligations associated with the contents of the international protection thus granted; (c) a permanent residence and work permit; (d) the issuance of identity and travel documents to whoever has been recognized as having the status of refugee and, where necessary, for those who benefit from subsidiary protection; (e) access to public employment services; (f) access to education, healthcare, housing, welfare benefits and social services, any rights recognized by the legislation which apply to people who are victims of gender-based violence, where appropriate, social security and integration programmes under the same conditions as Spaniards; (g) access to continuing or occupational training and to internships under the same conditions as Spaniards, as well as to the procedures used to recognize academic and professional diplomas and certificates, along with other official qualifications issued abroad; (h) freedom of movement; (i) access to integration programmes; (j) access to voluntary repatriation aid programmes; and (k) maintaining the family unit.

Suppose a person’s claim is rejected, not given leave to proceed, or the claim for international protection is rejected. In that case, it shall involve, as appropriate, return to the country of origin, refoulement, deportation, mandatory deportation from Spanish territory, or transfer to the territory of the state holding responsibility for examining the asylum claim of the person who has filed it, except where, according to Organic Act 4/2000, the interested party meets the requirements to remain in Spain temporarily or reside in the country, or where his or her leave of stay or residence in Spain is authorized due to the humanitarian reasons set forth in prevailing legislation.

Even though these rights have been established, the people who flee war, violence, and other disasters do not always manage to file a claim for asylum due to the numerous obstacles they encounter when doing so and their hindrances during the asylum process itself. These include the difficulty of reaching the European Union’s territory due to tight border controls, the lack of information about the possibility of claiming asylum, illegal refoulement, specific measures affecting particular groups, the current economic crisis, and not understanding the host country’s language and culture.

#### 1.5.3. Stages of the Asylum Procedure

**Figure 1:**
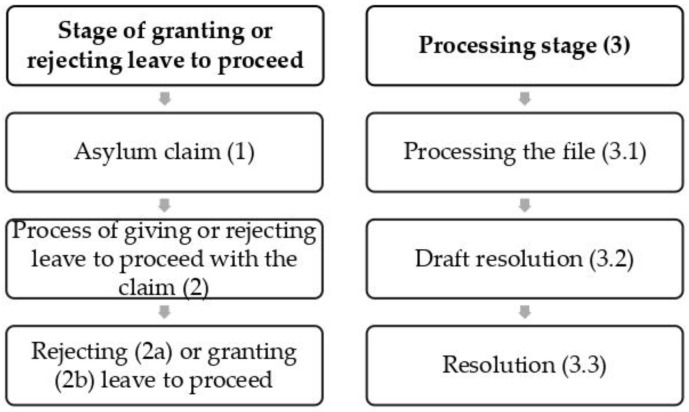
Stages of the asylum procedure in Spain, Based on CEAR [40].

The asylum procedure’s structure in Spain is essentially divided into two well-differentiated stages (Figure 1): granting or rejecting leave to proceed with the claim and the processing stage per se [40]. The first stage involves the claim for asylum (1), which consists of an interview with the person seeking asylum carried out by officials of OAR—Asylum and Refuge Office—or by other competent authorities, and the process of granting or rejecting leave to proceed with the claim (2), which consists of a preliminary examination to decide the claims that are worthy of processing: rejecting (2a) or granting (2b) leave to proceed. The second stage—processing (3)—consists of processing the file (3.1). In this stage, the OAR examines the file, studies the evidence, and issues a favorable or unfavorable report on the granting of asylum. A draft resolution is then drawn up (3.2), and the file and the report are considered by the Comisión Interministerial de Asilo y Refugio (CIAR—Inter-Ministerial Asylum and Refuge Commission). Finally, a resolution is drafted (3.3) by the Spanish Ministry of the Interior, provided it is in agreement with the CIAR’s findings. Should it disagree, the resolution is taken by the Cabinet. Two months after the claim is filed and granted leave to proceed, the OAR issues the refugee with the so-called “red card,” which authorizes him or her to remain in Spanish territory during the time the claim is being resolved and allows him or her to have a job, but only when six months have elapsed since the claim was filed [38,41].

Considering all of the above, the present study aims to draw a general profile of refugees’ and asylum seekers’ main characteristics in Spain and their level of well-being. More specifically, the parameters associated with their sociodemographic profile, linguistic knowledge, the social and institutional support they have received during the asylum process, and their well-being will be considered. We consider this to be a pivotal first step that provides useful information for further aid strategies. Knowing the profile of the asylum seeker and refugee in Spain, not only in terms of their sociodemographic characteristics but also in terms of their language skills, the support they receive, and their emotional well-being is fundamental in order to understand their situation better and to propose the design of strategies that will promote better use of the resources available to them and, above all, to protect their rights as human beings and ensure their emotional well-being and health.

## 2. Materials and Methods

### 2.1. Participants

The sample comprised 186 asylum-seekers, refugees, and people whose claim for asylum had been rejected residing in five Spanish regions: Andalusia, Catalonia, Madrid, Valencia, and the Basque Country. A majority of men amounted to 135 (73.8%) of the participants, as opposed to 48 women (26.2%). The participants’ age ranged from 18 to 62 years, with an average age of 31.7 years and a standard deviation of 9.8.

### 2.2. Instruments

An ad-hoc questionnaire was administered in this study to obtain the information needed to define refugees’ profiles in Spain. Said questionnaire was developed based on a bibliographic review, interviews with refugees, and a focus group. Prior to the questionnaire being administered, a pilot test of the questionnaire was conducted in a reception center in Valencia.

The questionnaire is comprised of 22 questions that assess the refugee’s profile. The questions are grouped under the following thematic blocks: demographic information, information on linguistic knowledge, and information on social and institutional support. The questionnaire was administered online and in hard copy. English, French, and Arabic versions were drawn up from the initial Spanish version.

#### 2.2.1. Sociodemographic Profile

The variables taken into consideration included: age (question 1), sex (question 2), marital status (question 3), dependents under their care (questions 4 and 5), educational level (question 6), incomplete studies (question 7), current employment situation (question 18), past employment situation (question 12), current self-perceived economic situation compared with the past (question 17), time spent in Spain (question 14), and reasons for leaving their country of origin (question 19).

#### 2.2.2. Linguistic Knowledge

The questionnaire asked about knowledge of different languages (question 9), which included Spanish, as well as the other languages the subjects spoke before reaching Spain (question 10). The participants had to specify their knowledge of all the languages on a five-point scale (1 being “I neither speak nor understand the language” and 5 being “I command the language both orally and in writing”). They were also asked about their native language (question 11).

#### 2.2.3. Social and Institutional Support

Social and institutional support issues were dealt with in the questionnaire in different questions, namely: question 15 (Do you have any friends or family members you can rely on when you have a problem? Of these, how many live in Spain?); question 16 (How frequently do you get in touch with family members or friends?), which they had to respond by measuring the frequency on a five-point Likert scale (from 1 “never” to 5 “very often”); question 21 (Have you received aid from any NGO or other institutions?); question 22 (What kind of aid have you received?), which included the following options: legal aid (right to asylum, laws), healthcare, search for work, training courses, information on and search for housing and others, and which the participants could mark as many as they deemed suitable; and question 35 (Indicate the ease or difficulty you have encountered with the following aspects related to your stay in Spain on a scale of 1 to 5 (1 being “very difficult” and 5 being “very easy”), 35.1 (Communicating), 35.2 (Making friends), 35.3 (Finding work), 35.4 (Finding housing), 35.5 (Having healthcare), 35.6 (Having legal aid), 35.7 (Having training courses), 35.8 (Having translation and interpretation services), and 35.9 (Having enough money).

#### 2.2.4. Well-Being

A standardized instrument, SPANE [42], was used to measure well-being. It is a questionnaire of 12 items, of which six are used to evaluate positive feelings (SPANE-P) and the other six to evaluate negative ones (SPANE-N). For each of these groups (positive or negative), there are three general items; for the positive feelings we find: positive, good, pleasant; for the negative ones: negative, bad, and unpleasant. Then there are the specific items for each of the groups. For the positive: happy; joyful; contented. For the negative: sad; afraid; angry. On the other hand, this questionnaire refers to the moment in which the subjects experienced these feelings. Specifically, it asks what the individual has done and experienced in the last four weeks; however, as the authors of the questionnaire point out [42], it is possible to adapt it to the time that best suits the study.

Apart from the positive and negative feelings, it is also possible to calculate the general score, that is, the affective balance (SPANE-B). Participants should respond according to a Likert-type format with five anchors (1 = Very rarely or never and 5 = Very often or always). This scale has obtained good results in terms of reliability and validity in the different contexts in which it has been used.

The SPANE scale has been translated into six different languages: Arabic, French, Hindi, Italian, Japanese, and Turkish. It has obtained good results in terms of reliability and convergent validity with other measures of emotions (PANAS), happiness (Fordyce Happiness Scale), and satisfaction with life (Satisfaction with Life Scale, SWLS) [42]. For this research, the questionnaire was translated into Spanish.

### 2.3. Procedure and Data Analysis

Contact was established with asylum-seekers, refugees, and people who had been refused asylum thanks to several entities and organizations’ collaboration. Although all the organizations that work to defend refugees were asked to participate in this study, only CEAR—Spanish Aid to Refugees Commission, the Mislata (Valencia) Refugee Reception Centre, the Seville Refugee Reception Centre, and the Red Cross took part.

The sample was obtained from 12 different places located in five Spanish regions (Andalusia, Catalonia, Madrid, Valencia, and the Basque Country). Its distribution is shown in Table 1.

The questionnaires were applied by two interviewers trained in the main working languages (they both had university studies in philology, translation, and interpretation) who had experience in immigration and intercultural mediation matters. The anonymity and voluntary nature of the data gathered was ensured at all times. A presentation of the study was given in different languages, which included Spanish, English, French, and Arabic. In such a presentation, it was emphasized that the data would be kept confidential and anonymous at all times and that their responses would in no way place their asylum-seeking procedure in jeopardy.

The statistical analysis of the data was carried out using the SPSS 24.0, IBM, New York, NY, USA. The main descriptive parameters of the variables under study were calculated to determine the profiles.

## 3. Results

In order to define the profile of the refugees included in our study, we focused on their sociodemographic characteristics (age, sex, marital status, dependents under their care, origin, educational level, economic and work situation, reasons for leaving their country of origin, time spent in Spain), their linguistic knowledge and the social and institutional support they had received (aid received and access to different services during the asylum process).

### 3.1. Sociodemographic Profile

#### 3.1.1. Age, Sex, Marital Status, and Dependents

A clear majority of men amounted to 135 (73.8%), among the interviewees, as opposed to 48 women (26.2%). The participants’ age ranged from 18 to 62 years old, with an average age of 31.7 and a standard deviation of 9.8. Concerning the interviewees’ marital status, most of them were single (58.9%). The percentage of married persons amounted to 35.6% of the sample, widows and widowers accounted for 3.3% of the sample, and separated and divorced persons accounted for 2.2% of the sample.

Regardless of their marital status, 80 of the 186 interviewed (40%) stated that they had dependents under their care. If we focus on the number of dependents they had under their care, 32.5% affirmed they had to care for two dependents, 25% stated they had to care for one dependent, 13.8% responded they had three dependents under their care, and another 13.8% stated they had four dependents. Regarding the rest, 11.3% said they had five dependents under their care, while 1.3% affirmed they had six dependents, another 1.3% stated they had eight, and the remaining 1.3% stated they had ten dependents under their care. Only 26 of these 80 people stated they lived with their dependents (or with some of them) in Spain.

#### 3.1.2. Origin

Regarding the region of origin of the participants, 46.8% came from Africa, 39.8% from the Middle East and North Africa, 9.9% from Asia, 1.8% from Eastern Europe, and 1.8% from America; specifically, Central and South America.

If the data is analyzed by sub-regions, most of the people were Africans (77 AS&R) from West Africa (Burkina Faso, Benin, Ivory Coast, Ghana, Guinea, Liberia, Mali, Nigeria, Sierra Leone, and Togo), which accounted for 67.5%, followed by Central Africa and the Great Lakes (Cameroon, Congo, Central African Republic and the Democratic Republic of Congo), which accounted for 24.7%, and East Africa and the Horn of Africa (Chad, Somalia, and Uganda), which accounted for 7.8%. As for the Asian region (15 AS&R), most of the people came from South Asia (Afghanistan, Bangladesh, Iran, and Pakistan), which accounted for 80%, followed by South-East Asia (Myanmar and Uzbekistan), which accounted for 13.3%, and East Asia (China), which accounted for 6.7%. Regarding those interviewed that came from America, 66.3% came from Columbia and Venezuela and 33.3% from Honduras. The refugees that came from Europe were from Belarus, the Russian Federation, and Ukraine. From those who came from the Middle East and North Africa, the majority (91.4%) came from the Middle East (Jordan, Syria, and Palestine) and the resting 8.6% from North Africa (Algeria, Egypt, Morocco, and Western Sahara). If we specifically look into the places of origin of the asylum-seekers and refugees surveyed, it can be seen that most of them come from Syria, which accounts for 26.9% of the sample, followed by Mali, which accounts for 10.8%, and Palestine, which accounts for 7%; 8.1% did not specify their place of origin.

#### 3.1.3. Educational Level

Regarding the educational level of the sample of asylum-seekers and refugees surveyed, 13.5% of the people who responded to this question (178) had no studies, as opposed to 15.2% who had completed primary school studies, 37.6% who had completed secondary school studies, 30.9% who had gone to university, and 2.8% who had completed post-graduate studies. Furthermore, a considerable number (143 people), amounting to 76.9% of the total number of people surveyed, had started studies they were unable to complete before leaving their country of origin.

#### 3.1.4. Economic and Work Situation

When the asylum-seekers and refugees were asked about their current economic situation compared to the past, most of them (46%) stated that it had worsened, while 26.9% said it had not changed, and 19.9% affirmed it had improved.

Regarding their current work situation, 90.6% affirmed they were unemployed, while 6.4% said they were in remunerated work, and 2.9% stated they did voluntary work.

Moreover, when asked about their occupation before leaving their country of origin, 89.78% of the participants said they were working, while 17.74% stated they were studying, and 11.29% affirmed they were working and studying at the same time.

#### 3.1.5. Reasons for Leaving Their Country of Origin

In response to the question on the reasons for leaving their country of origin, 68.7% of the people who responded to this question stated they had left the country because of a situation of conflict or war in the country, 22.1% said they had felt persecuted, and 9.2% affirmed they had left their country for economic reasons. The people who answered this question also referred to problems having to do with sexual orientation, imprisonment of family members, racism, oppression, and other reasons.

#### 3.1.6. Time Spent in Spain

The amount of time the AS&R had been living in Spain ranged from 0.33 months (10 days) to 360 months, which is equivalent to 30 years. The average time spent in Spain stood at 26.10 months (2.17 years), with a standard deviation of 38.28. The statistical trend indicates that the participants had most commonly been in Spain for between 4 and 9 months.

#### 3.1.7. Linguistic Knowledge

This study’s findings show that the most commonly spoken native language was Arabic, with 71 subjects, followed by Bambara with 16 subjects and Dyula with eight subjects.

The average knowledge of the spoken languages ranged between 4.07 and 4.49 (1 being “I neither speak nor understand the language” and 5 being “I command the language both orally and in writing”) with a standard deviation of between 0.91 and 1.21. Of the total of 186 asylum-seekers and refugees who responded to the questionnaire, 62 answered the question on the languages they spoke other than their native language and Spanish. The most commonly spoken language was English (25.64%), followed by French (19.37%), Arabic (17.95%), and Bambara (5.41%).

Regarding their knowledge of Spanish, it can be observed that the average level of knowledge of the 170 people who answered this question was 2.77 (1 being “I neither speak nor understand the language” and 5 being “I command the language both orally and in writing”) with a standard deviation of 1.10. If we now look into the percentages, 10.6% stated they neither spoke nor understood it, while 35.3% said they had basic knowledge, 31.2% affirmed they had an intermediate knowledge, 14.1% said they had advanced knowledge, and 8.8% affirmed they commanded the language both orally and in writing.

### 3.2. Social and Institutional Support

#### 3.2.1. Social Support

When asked the question: “Do you have any friends or family members you can rely on when you have a problem? How many?”, 55 people affirmed they could rely on friends and family members, 29.1% said they had one person they could rely on if they had problems, another 29.1% stated they could rely on two people, and 21.8% said they could rely on three people. Of the people who could rely on others, 70.5% of those who responded (44 people) stated that one or two of these people actually lived in Spain.

As regards the frequency with which they maintained contact with friends or family members, the average number of people with whom they maintained contact was 3.56, with a standard deviation of 4.92 (1 being “never” a 5 being “very often”). Of the people who responded to this question, the minimum number of friends or family members was 1, and the maximum was 30.

When asked about the frequency with which they maintained contact with their friends or family members, the average frequency obtained was 2.95 (1 being “never” and 5 being “very often”). Most of the asylum-seekers and refugees who took part in the study who answered this question affirmed they were sometimes in contact (46.3%), followed by 21.7% who affirmed they were frequently in contact and 15.4% who said they were never in contact with family members or friends.

#### 3.2.2. Institutional Support

If we focus on institutional support, that is to say, the aid provided by different NGOs and institutions to the asylum-seekers and refugees, it was found that 71.8% of the participants who responded to this question (177) stated they had received aid from an NGO or other institutions. When asked about the NGOs and institutions that had helped them, 62.14% said they had received aid from CEAR, followed by 15.71% who recognized they had received assistance from the Red Cross and 4.29% from the Refugee Reception Centres (CAR).

Concerning the specific kind of aid they had received, 85.4% of the AS&R stated they had received legal aid, 8.9% affirmed they had received healthcare, 3.2% said they had been helped to look for a job, and 1.3% affirmed they had received information about how to look for housing.

The asylum seekers and refugees likewise responded on the ease with which they had access to nine elements, namely: communicating, making friends, finding a job, finding housing, having legal aid, doing training courses, having translation and interpreting services, and having enough money. They also responded on a five-point scale (1 being “very difficult” and 5 being “very easy”) in this case. In general terms, access to most of the elements by the AS&R seems to have been relatively easy since the scores tend to be above 3, the halfway point. Nonetheless, three aspects seem to be difficult to gain access to (scores less than 3): work, money, and housing.

When the data is sorted by the ease of access, it was found that healthcare is ranked first, with an average of 3.82 and a standard deviation of 1.23. It is followed by gaining access to legal aid with an average score of 3.64 and a standard deviation of 1.21. These aspects are followed by the difficulty or ease of communicating, with an average score of 3.44 and a standard deviation of 1.37. The ease of doing training courses is ranked next, with an average score of 3.29 and a standard deviation of 1.35, followed by the ease or difficulty of making friends, which had an average score of 3.09 and a standard deviation of 1.34. Ranking fifth was the ease of gaining access to translation and interpreting services with an average score of 3.28 and a standard deviation of 1.32, which was followed by the ease of finding housing with an average score of 2.79 and a standard deviation of 1.48. Then the ease of having access to money is ranked next with an average score of 1.92 and a standard deviation of 1.22, and lastly, we find the ease of finding a job with an average score of 1.91 and a standard deviation of 1.40.

In regards to the question of having access to translation and interpreting services, it was revealed that 45% of the respondents considered it to be very easy (23.2%) or quite easy (23.2%), 23.9% considered that it was neither easy nor difficult, 18.1% considered it to be difficult, and 11.6% said it was very difficult. This broad range of responses led to the ease of gaining translation and interpreting being ranked fifth.

### 3.3. Well-Being

About the results obtained on the well-being of asylum seekers and refugees since they arrived in Spain with the SPANE scale, three differential scores were calculated: positive feelings (SPANE-P), negative feelings (SPANE-N), and emotional balance (SPANE-B). Table 2 shows the main descriptors in these dimensions.

Considering the responses obtained (Table 2), it seems that refugees present medium-high values in positive feelings (18.96 out of a range of 6 to 30) and medium scores in negative feelings (15.66 out of a range of 6 to 30).

In general, the respondents present more positive feelings (Mean = 18.96; DT = 6.01) than negative ones (Mean = 15.66; DT = 5.13), which implies a positive emotional balance (Mean = 3.29; DT = 5.67), although the average score obtained for emotional balance is quite far from the highest possible score (24).

Table 3 and Table 4 present the distribution of the refugee according to the type of score in positive affect (SPANE-P) and in negative affect (SPANE-N).

Likewise, the affective predominance in the SPANE-B was calculated (Table 5) considering, on the one hand, those persons in whom subtracting the positive feelings from the negative ones obtained a negative result, as opposed to those persons in whom performing such subtraction obtained a positive affective balance (positive affective predominance).

According to the results obtained, most refugees (71.3%) seem to present average scores in positive feelings, compared to 14.4% who present low positive feelings and another 14.4% who show high positive feelings.

Observing the values obtained in negative feelings in relation to the time from their arrival in Spain, it seems that more than half of the refugees (67.5%) present average scores in negative feelings, compared to 18.4%, who seem to present high negative feelings and 14.1% who present low negative feelings.

If we consider the emotional balance, most of the respondents (65.8%) seem to present a balance in which there is a predominance of positive feelings, compared to 34.2% in which negative feelings predominate (Table 5).

## 4. Discussion

The claim for asylum is vitally important for the person requesting it because, if granted, it invokes a system of legal protection and welfare benefits that allows the person to start a new life. This paper provides data regarding how it is a complicated and arduous process in which the person has to overcome a series of obstacles, starting with abandoning their country of origin and reaching the host country, the lack of information concerning the possibility of seeking asylum, the “border screening,” the illegal refoulement, specific measures against particular groups, the current economic crisis, and the lack of knowledge of the language and culture of the host country.

Once in the territory, states must ensure that applicants are informed, in a language they understand, of their rights and obligations when applying for asylum, as recommended by the United Nations High Commissioner for Refugees [1].

The refugee phenomenon has become increasingly prevalent, remaining in 2019 at a record high [1]. Therefore, it is essential to conduct studies that can help us gain a better understanding of these people’s profiles in order to put measures into place that would improve their situation and facilitate their integration in the host country.

In this light, this paper’s main objective consisted of determining the profile of asylum-seekers and refugees in Spain regarding their sociodemographic profiles, linguistic knowledge, the social and institutional support they have received during the asylum process, and their well-being. As commented in the limitations section, the sample of participants is limited and, therefore, so is the generalizability of its results. Nevertheless, our data suggest that the average profile of an asylum-seeker or refugee in Spain is a single male in his 30 s with no dependents, who has traveled alone, fled from conflict or war in his country of origin in Africa or the Middle East, who has been living in Spain for 2.17 years, and that had started high school or university education that he was unable to complete before he left his country of origin. He speaks English or French as a second language and has a basic or intermediate level of Spanish. Prior to his arrival in Spain, he has not usually had contact with interpreting services. He can barely count on family or friends; his economic situation has worsened since his arrival; he is unemployed and receives assistance from NGOs.

As far as institutional support is concerned, the area where AS&R receive by far the greatest assistance is legal aid, followed at a great distance by healthcare. Regarding the ease or difficulty of gaining access to different aspects, it can be seen that healthcare and legal aid are the easiest to access. On the other hand, finding a job, having money, and finding housing are the most difficult to access.

There does not seem to be any kind of consensus concerning translation and interpreting services, given that one part of the sample considers it to be easy, and the other finds it difficult to gain access to this service. This fact may perhaps be due to the sector’s lack of professionalization since most of the interpreting is done on an ad-hoc basis by family members and friends and not by trained professionals [43,44,45,46,47].

With regard to the results obtained on well-being with the SPANE scale, three differential scores were calculated: positive feelings (SPANE-P), negative feelings (SPANE-N), and emotional balance (SPANE-B). According to the responses obtained, the AS&R present medium-high values in positive feelings and medium scores in negative feelings. Therefore, in general, it seems possible to say that the refugees present more positive feelings than negative ones, which implies a positive emotional balance, although the average score obtained for emotional balance is quite far from the highest possible score. At this point, it should be pointed out that sometimes the AS&R responded to this question aloud since they needed help to answer the questionnaire. For this reason, their answers could be biased towards positive, as may have happened in other questions of the questionnaire. When trying to create a positive image of themselves, such as social desirability, they sometimes even marked their answers by their own beliefs about how they should be good people and the importance of having a prevalence of positive feelings.

### 4.1. Practical Implications

On the one hand, our sample’s sociodemographic profile tells us about the absence of women in this group. The causes of this fact should be studied in future research in order to elucidate this phenomenon better and to be able to provide the necessary and specific help in the different migratory points and to protect in particular women and unaccompanied minors, who are especially vulnerable within this already vulnerable group [1,8,11,12,48,49].

Concerning the institutional support AS&R receive, our data indicate that the most significant deficiency is in the difficulty of finding a job. Therefore, a practical implication of our data would be to try to strengthen this aspect of the institutional support they receive, since the vast majority of our participants were unemployed, which makes it impossible for them to gradually achieve the independence and autonomy they need, the normalization they require of their lives, and to build a life that satisfies their most basic needs, including those of emotional well-being.

As far as language skills are concerned, on the one hand, the vast majority of participants speak a minimum of two languages, many of them even speaking three or four. This fact could be pointing out a characteristic to be taken into account to find employment for some of these AS&R in intervention and integration programs.

Finally, concerning the emotional well-being of the AS&Rs, they present average levels of both positive and negative emotions. On the one hand, as already mentioned, this could be due to cultural values and social desirability before the interviewer. However, these data may also be reflecting that, despite having positive emotions related to the improvement of their life situation in comparison with their place of origin or with the journey to Spain, at the same time, the AS&R have a high load of negative emotions related on the one hand to the reality they have left behind and the possible traumas associated with it, as well as an uncertainty about their future. If we take into account that the great majority of those interviewed reported having very little social support, it seems important that plans aimed at alleviating the suffering of this group also consider, on the one hand, the detection of possible traumas and their treatment, and on the other hand, the provision of strategies and support to alleviate the heavy burden of uncertainty about their future. Further research would be necessary in order to better elucidate the reasons behind the positive and negative emotions of AS&R in Spain; a bigger sample than the one from the present study would allow for this. Nonetheless, our data highlights the importance of taking into consideration these variables, among which finding a job stands out as a key factor.

### 4.2. Limitations and Future Lines of Research

Even though this study attempts to gather refugees’ voices in Spain and that studies like this are needed to this end, it is not exempt from limitations. On the one hand, the sample’s representativeness is limited, particularly regarding its size and the various statuses of the persons under scrutiny. The sampling of the study has an impact on the generalizability of its results. Although data must be generalized with caution, the information provided is relevant for policy planning. At the same time, the methodology used allows for a profile of the participants in terms of the variables studied, but it does not allow for the elucidation of more complex relationships between them, such as causality. Nonetheless, we believe that the data provided are valuable and relevant and should be collected and considered. We hope that this study works as a first approach to the reality of this group so forgotten and in need of social conscience about their difficult situation, so that not only the responsible entities work for the welfare of refugees and their rights, but also the society that hosts them. Future research lines could extend the sample size to obtain more representative data at a national level, allowing for a more detailed study of the relationship between the variables here collected.

In addition to collecting more data from a broader sample, the results of this work could benefit from additional information provided by other agents involved (service providers, institution managers, and interpreters), allowing a methodological triangulation in order to obtain a much broader view of the reality of this phenomenon, which is undergoing constant change due to the rapid evolution of the world’s conflicts. For example, during the current global crisis caused by COVID-19, countries like Portugal and Italy have taken measures in order to regularize the situation of some of the AS&R [50]. These measures, however, have not been taken yet in Spain.

Finally, it would also be of interest to determine whether the differences found in the profile from our sample could affect other important social and personal variables like quality of life.

## 5. Conclusions

Most of the data provided herein are new since no other official research or studies have dealt with these issues in Spain. This fact makes it difficult to compare this paper’s findings with similar previous research on this matter and context. In this regard, the Oficina de Asilo y Refugio (Asylum and Refugee Office), the source on which CEAR’s reports are based, provides sociodemographic information on age, sex, and origin. However, these reports do not cover the areas dealt with in this paper, such as dependents under their care, educational level, economic and work situation, reasons for leaving their country of origin, time spent in Spain, linguistic knowledge, and social and institutional support, and well-being.

Due to all this, this study constitutes a preliminary approach aimed at taking a snapshot of the reality of refugees in Spain regarding their sociodemographic characteristics, the institutional and social support they receive, and their emotional well-being. However, it does provide some useful data that could be of use to improve the situation of such a vulnerable group by allowing the tailoring of intervention programs aimed to improve their situation and facilitate their well-being and the protection of their rights.

## Figures and Tables

**Table 1:** Location of the interviewed Refugees.

Centre	Frequency	Percentage
Cullera Migration Centre	18	9.7
Getafe Migration Centre	18	9.7
CEAR Basque country	34	18.3
CEAR Malaga	16	8.6
Seville Refugee Reception Centre	28	15.1
CEAR País Valencià	12	6.5
Bilbao Red Cross	3	1.6
Barcelona CCAR	15	8.1
Malaga Red Cross	6	3.2
Mislata Refugee Reception Centre	27	14.5
Mislata Football Championship	2	1.1
Africa Day, Nazaret	7	3.8
Total	186	100

Notes: CCAR = the Catalonian Aid to Refugees Commission; CEAR = Spanish Refugee Aid Commission.

**Table 2:** Main descriptive data of SPANE.

		SPANE-P	SPANE-N	SPANE-B
N	Valid	167	163	161
	Lost	19	23	25
Mean		18.96	15.66	3.29
SD		6.08	5.67	8.30
Minimum		2.00	1.00	−24.00
Maximum		30.00	30.00	24.00
Range		6 a 30	6 a 30	−24 a 24

Note: SD = Standard deviation.

**Table 3:** Distribution of the sample according to the type of score in SPANE-P.

		Frequency	Percentage
Valid	Low	24	14.4
	Medium	119	71.3
	High	24	14.4
	Total	167	100.0
Lost	System	19	
Total		186	

**Table 4:** Distribution of the sample according to the type of score in SPANE-N.

		Frequency	Percentage
Valid	Low	23	14.1
	Medium	110	67.5
	High	30	18.4
	Total	163	100.0
Lost	System	23	
Total		186	

**Table 5:** Distribution of the sample according to the type of score in SPANE-B.

		Frequency	Percentage
Valid	Predominantly negative affect	55	34.2
	Predominantly positive affect	106	65.8
	Total	161	100.0
Lost	System	25	
Total		186	
